# How animals distribute themselves in space: variable energy landscapes

**DOI:** 10.1186/s12983-017-0219-8

**Published:** 2017-07-05

**Authors:** Juan F. Masello, Akiko Kato, Julia Sommerfeld, Thomas Mattern, Petra Quillfeldt

**Affiliations:** 1 Department of Animal Ecology & Systematics, Justus Liebig University Giessen, Heinrich-Buff-Ring 26, D-35392 Giessen, Germany; 2 Centre d’Etudes Biologiques de Chizé, UMR7372 CNRS-Université La Rochelle, 79360 Villiers en Bois, France

**Keywords:** Energetic costs, Energy landscape, Foraging effort, Foraging strategy, Landscape of fear, Ecological mechanism, Movement ecology, Non-lethal effects of predation, Tri-axial acceleration, Variable costs of foraging

## Abstract

**Background:**

Foraging efficiency determines whether animals will be able to raise healthy broods, maintain their own condition, avoid predators and ultimately increase their fitness. Using accelerometers and GPS loggers, features of the habitat and the way animals deal with variable conditions can be translated into energetic costs of movement, which, in turn, can be translated to energy landscapes.We investigated energy landscapes in Gentoo Penguins *Pygoscelis papua* from two colonies at New Island, Falkland/Malvinas Islands.

**Results:**

In our study, the marine areas used by the penguins, parameters of dive depth and the proportion of pelagic and benthic dives varied both between years and colonies. As a consequence, the energy landscapes also varied between the years, and we discuss how this was related to differences in food availability, which were also reflected in differences in carbon and nitrogen stable isotope values and isotopic niche metrics. In the second year, the energy landscape was characterized by lower foraging costs per energy gain, and breeding success was also higher in this year. Additionally, an area around three South American Fur Seal *Arctocephalus australis* colonies was never used.

**Conclusions:**

These results confirm that energy landscapes vary in time and that the seabirds forage in areas of the energy landscapes that result in minimized energetic costs. Thus, our results support the view of energy landscapes and fear of predation as mechanisms underlying animal foraging behaviour. Furthermore, we show that energy landscapes are useful in linking energy gain and variable energy costs of foraging to breeding success.

**Electronic supplementary material:**

The online version of this article (doi:10.1186/s12983-017-0219-8) contains supplementary material, which is available to authorized users.

## Background

Animals do not distribute themselves randomly. An extensive literature on wild animal movements and habitat use shows that some locations are highly used, while other nearby locations are avoided [1–6]. Understanding the behavioural decisions that makes a place a foraging ‘hot-spot’ as compared to a corridor or even a no-go area will be crucial for securing safe spaces for wild animals facing expanding human influence [7] and climate change [8]. Optimal foraging theory [9, 10] predicts that animals will select patches abundant in resources where the gain per unit cost is high. Any unnecessarily extensive movements might increase the risk of predation, and thus, predator avoidance also influences the movements of many animals [5, 11, 12].

In addition to the description of the movement of organisms (e.g. [13]), it is important to consider movements in the context of ecological factors [5, 14–16]. Foraging costs have usually been investigated in terms of time, energy gained or energy consumed [17–19]. However, even minor landscape features may directly affect animal movements by imposing considerable energy barriers on travel [7]. Likewise, the degree of variation in the landscape will account for variable energy cost of movements [20], which can be translated into an energy landscape for animals foraging in it [21, 22]. Consequently, in landscapes where resources are not distributed in a way that resembles the energy landscape, animals will forage in areas of the energy landscape that result in minimized costs and maximised net energetic gain [21]. This prediction has been supported by studies that investigated foraging movements through energy landscapes using animal-attached devices to derive the energetic costs of foraging [7, 21–24]. In marine environments or “seascapes”, oceanographic conditions and currents vary over time related to oceanographic cycles and climate change [25–30], resulting in changes in food availability and distribution and thus, in energy landscapes. Such temporal changes of energy landscapes (between or within years) and their consequences on animal behaviour have not been investigated to date. Filling such a gap in our knowledge is particularly relevant in the context of climate change.

Seabirds have evolved a multitude of foraging strategies in order to successfully prey on marine food, such as species-specific preferences of prey or the use of open-ocean versus coastal habitats [16, 31, 32]. During the breeding season, seabirds are central-place foragers, exploiting resources within a given range around their colonies or nests [18, 33, 34]. In a previous study, we investigated simultaneous ecological segregation among species and colonies of a diving seabird assemblage, sharing a sector of the south-western Atlantic Ocean during the breeding season [5]. In that study, we deployed GPS-temperature-depth (GPS-TD) loggers on Gentoo, Rockhopper, and Magellanic penguins (*Pygoscelis papua*, *Eudyptes chrysocome*, *Spheniscus magellanicus*), and Imperial Shags (*Phalacrocorax atriceps*) breeding at New Island, Falkland / Malvinas Islands, during the breeding season. Because the studied seabird colonies at New Island were much closer to each other (2–7 km) than the average foraging range of the species (9–27 km), we expected large overlaps among the foraging areas. However, we found little, if any, overlap due to strong spatial and temporal segregation [5]. Particularly striking, we observed strong differences in foraging areas, diving depth, time of foraging and prey choice among birds of the same species, breeding in different colonies at the same island [5]. We concluded that the observed differences were most likely caused by optimal foraging of individuals in relation to habitat differences on a local scale, leading to a complex pattern of interactions with environmental covariates, combined with avoidance of predation [5]. Such a flexible foraging strategy was also observed in Gentoo Penguins from Antarctica, where differences were found among years [29, 35]. Flexible foraging habits would provide a buffer against changes in prey availability [29].

In the present study, we investigated the mechanisms behind the flexible foraging strategies in Gentoo Penguins. During two different years, using two colonies of Gentoo Penguins that previously showed strong spatial and temporal segregation [5], and GPS and tri-axial acceleration data for the calculation of energetic costs of movement [21], we aim to show that 1) energy landscapes vary in time (e.g. between breeding seasons) resembling the interaction between foraging effort and prey availability, 2) the seabirds will forage in areas of the energy landscapes that result in minimized energetic costs, 3) as central-place foragers are constraint in the area where they can forage, temporal changes in the energy landscape and associated changes in energy costs of foraging will affect the breeding success.

## Methods

### Study site and species

**Fig. 1:**
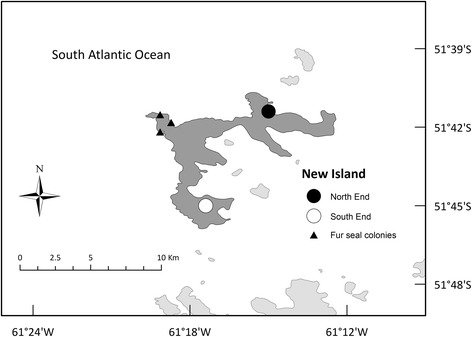
Location of the Gentoo Penguin *Pygoscelis papua* colonies studied. New Island (in *dark grey*) is located in the Falkland Islands/Islas Malvinas, Southwestern Atlantic. South American Fur seal *Arctocephalus australis* colonies are indicated with black triangles. See bathymetric map in Additional File 1: Figure S1

The study was conducted at New Island Nature Reserve in the Falkland Islands / Islas Malvinas, south-western Atlantic Ocean [36, 37]. At the continental slope, the Falkland Current generates a strong upwelling of productive Sub-Antarctic superficial water ([37] and references therein). This area of increased productivity attracts many seabird species, 13 of which breed in colonies distributed over New I. [38]. Among them is the Gentoo Penguin, which we investigated in two breeding colonies: one at the North End (51° 41.402′ S 61° 15.003′ W), and one at the South End (51° 44.677′ S 61°17.683′ W; Fig. 1) of New Island.

In a previous study, we found complete spatial segregation between these two colonies of Gentoo Penguins, regardless of their proximity (7 km apart), during the studied breeding season (chick guard 2008) [5]. Our study also showed that Gentoo Penguins started foraging very close (from 0.4 to 2.5 km) to the breeding colonies [5]. Gentoo Penguins have been found to be neritic foragers during the breeding season and among the main avian benthic consumers of the sub-Antarctic area, their diet varying greatly between locations and in time [32, 39]. Miller et al. [29] and Handley et al. [40] found that the prey of Gentoo Penguins comprised mainly benthic prey but regularly included pelagic prey. An earlier study of Gentoo Penguins at New I. [41, 42] was in line with these findings, as the diet comprised mainly lobster krill (*Munida gregaria*; 56%), followed by both benthic and pelagic fish (main items: *Micromesistius* sp., Nototheniidae and Perciformes; 34%) and squid (mainly *Gonatus antarcticus*; 9%) in 1986/87. For the North End colony at New I., Clausen et al. [43] found that Gentoo Penguins foraged mainly on pelagic prey (*Sprattus fuegensis*). In East Falkland, the principal prey items during chick guard were rock cod *Patagonotothen* spp. (78% in 2012), and Patagonian longfin squid (*Loligo gahi*) (7% in 2012) [40].

### Instrumentation and fieldwork procedures

GPS-temperature-depth (GPS-TD; earth & Ocean Technologies, Kiel, Germany) and micro tri-axial accelerometer (Axy; TechoSmArt Europe, Rome, Italy) loggers were simultaneously deployed on 32 Gentoo Penguins from the South End and North End colonies during chick guard (December) in 2013 and 2014 (Table 1). We were not allowed to work on the North End colony during 2013 due to the activities of a film crew. No loggers were deployed in days of bad weather conditions in order to ensure an effective protection of the chicks and the adult birds. Birds were captured mostly by hand, in the vicinity of their nests, with the occasional help of a hook attached to a rod [44]. Chicks were also captured to protect them from predators like Brown Skuas *Catharacta antarctica* and Striated Caracaras *Phalcoboenus australis* during the handling of the adult. Handling time was kept to a minimum, mostly below 15 min and always below 20 min. Extreme care was taken to minimize stress to the captured birds, with the head covered during handling in order to minimize the risk of adults regurgitating. During this procedure no great signs of stress were apparent: none of the birds regurgitated. The attachment of the loggers on the adult penguin was carried out using adhesive Tesa® 4651 tape as described by Wilson et al. [45]. Both loggers (GPS-TD: 75 to 145 g; Axy: 19 g) represent a maximum of 2.5% of the adult body mass (mean 6459 ± 172 g, *n* = 16) [5]. In a previous study [46], we showed that handling and short-term logger attachments like the ones here carried out showed limited effect on the behaviour and physiology of the birds. Other studies have also found no negative effects of similar GPS-loggers in the foraging behaviour or the breeding success of the birds [47–50]. GPS-TD loggers recorded detailed position (longitude, latitude; sampling interval: 5 min), dive depth (resolution: 3.5 cm; sampling interval: 1 s), and time of day. While at sea, GPS functionality was pressure controlled so as to attempt to obtain a GPS fix upon resurfacing from dives. The Axy loggers recorded acceleration (sampling interval: 50 Hz) measured in three directions (x, y, z, i.e. surge, sway, heave) (e.g. [51]). After the deployment procedure and immediately before the release of the adult bird, chicks were returned to the nest. The adults were released some 20 m from their nests. All birds returned to their nests and attended their chicks shortly after being released.

**Table 1:** Parameters of foraging trips used for the calculations of energy landscapes. The data correspond to Gentoo Penguin *Pygoscelis papua* breeding at New Island (Falkland/Malvinas Is.), during chick guard (December) in 2013 and 2014. Only the first foraging trip of each individual was included in the calculations in order to avoid individuals with more than one trip having more weight in the analyses

	2013	2014	
	South End	South End	North End
Individuals tagged	16	8	8
Number of complete data sets obtained (first foraging trips)	13	4	6
Trip length [km]	131.1 ± 59.1 (67.0–281.7)	92.7 ± 64.7 (24.1–169.4)	56.9 ± 13.7 (33.8–75.5)
t-test between seasons	*t =* 1.113	*P =* 0.283	
Mann-Whitney Rank Test between colonies		*T =* 24.000	*P =* 0.749
Maximum distance from colony [km]	69.1 ± 9.8 (51.3–87.6)	49.6 ± 33.0 (13.8–89.3)	33.3 ± 17.3 (15.3–60.1)
Mann-Whitney Rank Test between seasons	*T =* 26.000	*P =* 0.282	
t-test between colonies		*t =* −1.038	*P =* 0.330
Trip duration [min]	1811.5 ± 754.4 (770.6–2965.1)	1636.6 ± 1162.8 (320.7–3066.6)	1183.0 ± 353.4 (798.2–1650.8)
t-test between seasons	*t =* 0.129	*P =* 0.725	
Mann-Whitney Rank Test between colonies		*T =* 25.000	*P =* 0.610
Start time of foraging (local time)	07:14:53 ± 06:14:24 (02:12:13–19:16:23)	15:07:12 ± 04:50:53 (08:10:50–18:38:50)	10:22:05 ± 08:05:17 (02:47:49–20:26:48)
Mann-Whitney Rank Test between seasons	*T =* 54.000	*P =* **0.048**	
t-test between colonies		*t =* 1.044	*P =* 0.327

Sample sizes vary with respect to deployments, as not all parameters could be calculated for all individuals, mainly due to some batteries running out before the finalization of an ongoing trip. Statistically significant values are marked bold

The birds were recaptured in the vicinity of their nests after 2 to 12 days (median: 5 d) of logger deployment. All birds were recaptured and loggers recovered except in one case. Despite intensive efforts, we were not able to recapture one bird tagged in the South End colony in December 2014. It may be possible that the penguin abandoned the nest or that it was predated, as several Southern Sea Lions *Otaria flavescens* were intensively hunting at the penguin landing place during the deployment period. We observed several cases of Gentoo Penguin predation by sea lions while waiting for our tagged birds to return to the colony. Surprisingly, the two chicks belonging to the nest with the missing penguin developed normally, suggesting that they were adequately provisioned by the remaining parent. In any case, the unrecovered device was lost, at latest, during the natural moulting period (shortly after the breeding season) preventing any long-term consequences for the bird.

After logger recovery, the penguins were released as described above. All birds returned to their nests and attended their chicks shortly after being released except in one case. In this instance, the adult penguin took longer than usual to return to its nest and two Striated Caracaras predated the two chicks. No other cases of nest desertion were recorded and all chicks survived at least until the starting of the crèche period, a time when we were not able to identify individual chicks anymore.

### Spatial and temporal data

From 32 deployments in this study, we obtained 23 complete sets of tri-axial acceleration and GPS data, comprising location, time, and dive depth, which we used in the following analyses (Tables 1 and 2). Failures to produce complete data sets were due to 1) three GPS-TD loggers fully damaged by salt water reaching the electronic components, 2) two broken GPS antennas, and 3) four batteries that were unexpectedly depleted before the end of the first foraging trips. In 2013, seven Axy loggers were damaged by salt water but the data could be recovered. In 2014, all Axy loggers were recovered without any damage, as the logger coating was purposely reinforced by TechoSmArt and, additionally, the units were placed inside a tightly closed finger of a lab glove and then inside a heat-shrink tubing before deployment.

**Table 2:** Dive parameters used for the calculations of energy landscapes corresponding to Gentoo Penguin *Pygoscelis papua.* The study was conducted on penguins breeding at New Island (Falkland/Malvinas Is.), during chick guard (December) in 2013 and 2014. Only the first foraging trip of each individual was included in the calculations in order to avoid individuals with more than one trip having more weight in the analyses. For sample sizes see Table 1. For means, ranges are given in brackets, while for medians 75 and 25% quartiles are given

	2013	2014	
	South End	South End	North End
Maximum dive depth [m]	188.3	178.2	156.3
Mean number of dives per foraging trip (MND)	298 (176–674)	265 (81–648)	280 (192–343)
Mann-Whitney Rank Test between seasons	*T* = 50.000	*P* = 0.405	
Mann-Whitney Rank Test between colonies		*T* = 36.000	*P* = 0.445
Mean dive duration (DD), benthic dives [s]	166 (112–215)	175 (145–244)	180 (125–213)
Mann-Whitney Rank Test between seasons	*T* = 96	***P*** **= 0.002**	
Mann-Whitney Rank Test between colonies		*t* = 0.367	*P* = 0.721
Mean dive duration (DD), pelagic dives [s]	109 (87–158)	118 (112–140)	123 (108–146)
Mann-Whitney Rank Test between seasons	*t* = −1.610	*P* = 0.126	
Mann-Whitney Rank Test between colonies		*t* = −0.409	*P* = 0.690
Median dive event maximum depth [m]	21.9 (8.0–97.1)	45.1 (14.2–93.2)	45.2 (16.0–91.2)
Mann-Whitney Rank Test between seasons	*T* = 54,929,247.5	***P*** **< 0.001**	
Mann-Whitney Rank Test between colonies		*T* = 34,821,241.0	*P* = 0.985
Median dive depth of pelagic dives [m]	15.8 (6.3–77.0)	12.7 (5.8–41.2)	21.1 (9.2–48.9)
Mann-Whitney Rank Test between seasons	*T* = 1,203,123.5	***P*** **< 0.001**	
Mann-Whitney Rank Test between colonies		*T* = 834,201.5	***P*** **< 0.001**
Mean proportion of benthic dives (pBD) [%]	24 (10–40)	54 (22–72)	48 (30–76)
Mean proportion of pelagic dives (pPD) [%]	76 (61–90)	46 (27–78)	52 (24–70)
t-test between seasons	*t =* −3.828	***P =*** **0.002**	
t-test between colonies		*t =* −0.426	*P =* 0.678
Minimum benthic bottom time (mBBT) [s]	2	3	2

Statistically significant values are marked bold

As in previous studies (e.g. [5]), we defined foraging trips from the time when the birds departed from the colony to the sea until returning to the colony. Bathymetry data were obtained from the global sea floor topography from satellite altimetry and ship depth soundings (Global Topography; Additional file 1: Figure S1) [52] available at [53]. Positional data obtained from GPS-TD-loggers were used to plot and analyse the trips performed by the birds in ArcGIS 9.3 (ESRI, Redlands, USA). Trip length was calculated as the total cumulative linear distance between all positional fixes along the foraging trip, outside of the colony. For each trip, the maximum distance from the colony was calculated as the linear grand circle distance between the furthest point of the plotted trip and the geographical coordinates of the departure colony, determined by GPS. Trip duration was determined as the time elapsed between departure and return from the colony. Foraging dives were identified using purpose-written software in Matlab (The Mathworks Inc., Nattick, USA) and purpose-written script for IGOR Pro 6.3.7.2 (WaveMetrics, Lake Oswego, USA). Following Mattern et al. (2007) dive events could only be accepted when depths >3 m were reached. The bottom phase was defined as a period of the dive with little vertical undulation following a steady descent and before a steady ascent back to the surface [50, 54]. The maximum depth (in m) reached during a dive event (hereafter event maximum depth), and the number of dive events during a particular foraging trip were also calculated (Table 2). For each dive, we calculated a geographical position either by using the half way point between GPS fixes recorded immediately before and after the dive, or by calculating the relative position along a linear interpolated line between the last fix obtained and before the first fix after the dive occurred based on the time the dive occurred relative to these fixes.

As Gentoo Penguins were found to take both benthic and pelagic prey at the Falkland Islands [5, 40], the foraging dives performed by the individuals were split in benthic and pelagic ones for further analyses. This was done by calculating an index of benthic diving behaviour developed by Tremblay and Cherel [54]. This method assumes that benthic divers dive serially to a specific depth, and therefore consecutive dives reach the same depth zone. These are called intra-depth zone (IDZ) dives [54]. As in previous studies, the IDZ was defined as the depth ± 10% of the maximum depth reached by the preceding dive [16, 55]. During the current study, Gentoo Penguins performed a varying proportion of benthic and pelagic dives, which was taken into account in the following analyses (Table 2). As the inspection of histograms showed that the data for pelagic dives was left shifted, the median dive depth per colony per year was used for further calculations involving pelagic dives (Table 2; see Additional file 1: Figure S2; see also ‘Calculation of energy’). The geographical location of benthic and pelagic dives was checked in order to detect any potential bias in the distribution of the data. Benthic and pelagic dives were distributed evenly in the same depth areas of the ocean around New I. (see Additional file 1: Figures S3, S4). We also calculated the mean number of dives performed during the foraging trips (Table 1). In all calculations, only the first foraging trip of each individual was included in order to avoid individuals with more than one trip having more weight in the data. In a previous study [5], we found that the Gentoo Penguin from New I. showed no sexual differences in foraging behaviour parameters. Therefore, in this study, we pooled the data of males and females.

The nonparametric fixed kernel density estimator was used to determine the 20, 40, 60 and 80% density contour areas (estimated foraging range) [56] of dive locations (i.e. GPS position at the onset of a dive event). Kernel densities indicate the places in a foraging trip where birds spent most of their time [56]. Hawth’s Analysis Tools [57] in ArcGIS 9.3 were used to estimate a fixed kernel density using the quartic approximation of a true Gaussian kernel function [57]. GPS data-points at the colonies were excluded in order to avoid an overestimation of their importance.

When normality and equal variance tests passed (all *P* > 0.05), we used t-tests implemented in R to test for differences between colonies and seasons on the calculated trip and dive parameters (Tables 1 and 2) [58]. In cases where normality and equality of variance were not satisfied (*P* < 0.05), we used Mann-Whitney rank sum tests in order to investigate differences.

### Calculation of energy

Using a purpose-written script for IGOR Pro 6.3.7.2 (WaveMetrics, Lake Oswego, USA) and tri-axial acceleration data from Axy accelerometers, we calculated the Overall Dynamic Body Acceleration (ODBA) for all first foraging trips and individuals. ODBA is a linear proxy for metabolic energy that can be further converted into energy expenditure (e.g. [51, 59–63] but see [64]). ODBA (expressed as gravitational force *g*) was calculated as described in Wilson et al. [21]. We used the sum of the absolute values of dynamic acceleration from each of the three spatial axes (i.e. surge, sway, and heave) after subtracting the static acceleration (= smoothed acceleration) from the raw acceleration values [21]:

1 $$ \mathrm{ODBA}=\left| Ax\right|+\left| Ay\right|+\left| Az\right| $$

A_x_, A_y_ and A_z_ are the derived dynamic accelerations at any point in time corresponding to the three orthogonal axes.

The sum of ODBA during dives was related to the maximum dive depth (see Additional file 1: Figures S5–S10). However, a general additive model (GAM; see Additional file 1: Table S1) revealed that this relationship differed between studied years, colonies, and between benthic and pelagic dives. Thus, the regressions with the best fit were determined for the different combination of years, colonies and dive types in SigmaPlot 10.0 (Systat Software, San Jose, USA; see Additional file 1: Table S2; Figures S5–S10). We used the regressions between the sum of ODBA during the dive of the deployed penguins and the maximum dive depth (see Additional file 1: Table S2), together with the bathymetric data points from the Global Topography [52] to calculate benthic ODBAs for a grid of the marine area around New I. (approximately 100 km around the island; *n* = 26,196) separate for each colony and season. For the pelagic ODBAs, we used the corresponding regressions (see Additional file 1: Table S2) and the median dive depth per colony per year (Table 1; see ‘Analyses of spatial and temporal data’ for method validation).

The distance between each point in the marine area grid around New I. for which bathymetric data were available (see Additional file 1: Figure S1) and the Gentoo Penguin breeding colonies on New I. was calculated with the Hawth’s Analysis Tools [57] in ArcGIS 9.3. Using this distance and the mean swimming speed previously calculated for Gentoo Penguins (2.3 m s^−1^) [65], we were able to calculate the travel time needed for the birds to reach each of the 26,196 locations around New I. for which bathymetric data were available. The travel time (TT, in s), and their minimum metabolic cost of transportation (16.1 W kg^−1^) [65], allowed us subsequently to calculate the minimum cost of travelling (CT, in J kg^−1^) to each location:

2 $$ \mathrm{CT}=\mathrm{TT}\ast 16.1\mathrm{W}\kern0.5em {\mathrm{kg}}^{\hbox{-} 1} $$

Recent research demonstrated a linear relationship between ODBA and metabolic rate in all species examined to date (summarised in [21]; but see also [66]). Halsey et al. [61] investigated the relationship between the rate of oxygen consumption V_o_ (in ml min^−1^; an indirect measure of energy expenditure) and ODBA for 10 different species including Magellanic and Rockhopper penguins. The robust results obtained (*R* ^*2*^ = 0.99) allowed Halsey et al. [61] to propose a relationships between the species mean body mass (BM) and both the slope and intercept of the predictive relationships for all 10 species (including the two penguin species; *P* < 0.001 in all cases): intercept, y = 2.75 * BM^0.73^ slope y = 3.52 * BM^0.94^. Thus, following Halsey et al. [61], we first calculated:

3 $$ {\mathrm{V}}_{\mathrm{o}}=10.78+\mathrm{ODBA}\ast 20.45 $$

Although some inter-species variation can be observed in the analysis by Halsey et al. [61], the relationship for both penguin species is quite similar, allowing us to safely estimate a relationship between V_o_ and ODBA in Gentoo Penguins using the calculation method proposed by these authors.

In order to convert the uptake of 1 l of oxygen into energy expenditure we used the mean value of the oxidative catabolism of lipids, glucose and protein provided by Heldmaier et al. [67] (20 kJ), such that 1 ml O_2_/min equals 0.333 J s^−1^. To derive the mass-specific power (MP, in J kg^−1^ s^−1^) [21], the energy expenditure was divided by the mean weight of Gentoo Penguins (6.5 kg) [5]:

4 $$ {\mathrm{MP}=\mathrm{V}}_{\mathrm{o}}\ast 0.333/6.5\mathrm{kg} $$

The MP (4) can be calculated for each bathymetric data point in the grid of the marine area around New I. separately for benthic dives (MP_benthic_, based on bathymetric depth) and pelagic dives (MP_pelagic,_ based on the median dive depth during pelagic dives).

Subsequently, we calculated the MP for the grid of the marine area around New I. (see Additional file 1: Figure S1) and for both colonies and years, based on the mean number of dives per foraging trip (MND) and mean dive duration (DD, duration in s of the dive event; Table 2), assuming a gradient of bottom depths from 3 m to the maximum depth (= bathymetric depth), for benthic and pelagic dives as follows:

5 $$ {\mathrm{MP}}_{\mathrm{MND}\ \mathrm{benthic}}{=\mathrm{DD}}_{\mathrm{benthic}} \ast {\left({\mathrm{MP}}_{\mathrm{benthic}\ \left(3\ \mathrm{m}\ \mathrm{depth}\right)}+{\mathrm{MP}}_{\mathrm{benthic}}\right)}^{\ast}\mathrm{MND}/2\ast \mathrm{pBD} $$

6 $$ {\mathrm{MP}}_{\mathrm{MND}\ \mathrm{pelagic}}{=\mathrm{DD}}_{\mathrm{pelagic}} \ast {\left({\mathrm{MP}}_{\mathrm{pelagic}\ \left(3\ \mathrm{m}\ \mathrm{depth}\right)}+{\mathrm{MP}}_{\mathrm{pelagic}}\right)}^{\ast}\mathrm{MND}/2 \ast \mathrm{pPD} $$

where pBD is the mean proportion of benthic dives and pPD the mean proportion of pelagic dives (Table 2). These parameters together with previous calculations of the cost of travelling (CT), allowed us to calculate the total cost of foraging (TCF, in J kg^−1^) as:

7 $$ {\mathrm{TCF}=\mathrm{MP}}_{\mathrm{MND}\ \mathrm{benthic}}{+\mathrm{MP}}_{\mathrm{MND}\ \mathrm{pelagic}}+\mathrm{CT}\ast 2 $$

In order to build energy landscapes that also take into account the energy gained during foraging, we calculated bottom times (duration in s of bottom dive phase) and minimum benthic bottom times (mBBT; Table 1). The bottom times from the first foraging trip of each individual showed a relationship with maximum dive depth. This relationship also differed between studied years, colonies and between benthic and pelagic dives (GAM; see Additional file 1: Table S3). The regressions with the best fit were again determined for the different combination of years, colonies and dive types in SigmaPlot 10.0 (Systat Software, San Jose, USA; see Additional file 1: Table S4; Figures S11–S16). The regressions between bottom time and maximum dive depth (see Additional file 1: Table S4), together with bathymetric data [52] allowed us to calculate the sum of benthic bottom time (BBT) for each bathymetric point (see Additional file 1: Figure S1), separately for each colony and year. The minimum benthic bottom time for each colony and year is shown in Table 1. For pelagic bottom times (PBT), we used the corresponding regressions (see Additional file 1: Table S4) and the median dive depth per colony per year (Table 2; see ‘Analyses of spatial and temporal data’ for method validation). For the calculation of the total bottom time (TBT, in s), we took into account that the birds start diving close to the colony (as also found in [5]) and increase dive depth while gaining distance. A mean is calculated and the mean multiplied per the mean number of dives:

8 $$ \mathrm{TBT}=\left(\mathrm{mBBT}+\mathrm{BBT}\right)/2 \ast \mathrm{MND}\ast \mathrm{pBD}+\mathrm{PBT}\ast \mathrm{MND}\ast \mathrm{pPD} $$

Finally, dividing TCF (7) by TBT (8) we were able to calculated the total relative cost (TRC, in J kg^−1^ s^−1^) as the total cost of foraging (TCF; diving plus commuting) relative to the total bottom time (TBT). Using TRC values calculated for the grid of the marine area around New I. for which bathymetric data was available (*n* = 26,196; see Additional file 1: Figure S1), we constructed the energy landscape by applying the Inverse Distance Weighted (IDW) interpolation in ArcGIS 9.3 to the resulting data grid. The IDW interpolation was chosen as 1) a large set of sample values was available, and 2) the sample data points represented the minimum and maximum values in our surface [68]. Thus, the energy landscapes here presented are based on the bathymetry of the area and the total cost of foraging (diving plus commuting) relative to the bottom time (in J kg^−1^ s^−1^), and take into account the different proportion of benthic and pelagic dives carried out by the penguins in each studied colony and year.

### Stable isotope niche analysis

We analysed carbon (δ^13^C) and nitrogen (δ^15^N) stable isotope values of chick feather samples as a marker of breeding season foraging ecology. Feathers were sampled when the chicks were around 2 months old (February), ensuring that the feathers were grown during the time of deployment of the loggers (December). Twenty feathers were analysed from each colony and year except for the North End colony in 2014, for which we analysed 18 samples. Carbon and nitrogen isotope analyses were carried out on 0.65–0.75 mg sample aliquots, weighed into tin cups. Carbon and nitrogen isotope ratios were measured simultaneously by continuous-flow isotope ratio mass spectrometry (CF-IRMS) at the UC Davis Stable Isotope Facility, using a PDZ Europa ANCA-GSL elemental analyser interfaced to a PDZ Europa 20–20 isotope ratio mass spectrometer (Sercon Ltd., Cheshire, UK). Laboratory standard measurements have been previously calibrated against NIST Standard Reference Materials indicated a standard deviation is 0.2‰ for ^13^C and 0.3‰ for ^15^N. Stable isotope ratios were expressed in δ notation as parts per thousand (‰) deviation from the international standards V-PeeDee Belemnite for δ^13^C and to atmospheric N_2_ for δ^15^N.

The isotopic niches of birds from the two colonies were calculated using SIBER (Stable Isotope Bayesian Ellipses in R) [69]. In this analysis, the location of the centroid (LOC) indicates where the niche is centred in isotope space. A Bayesian approach based on multivariate ellipse metrics was used to calculate the standard ellipse area (SEA), which represents the core isotope niche width as described by Jackson et al. (2011). To describe the spread of the data points, parameters proposed by Layman et al. [70] were calculated. As proxies of intra-population trophic diversity, the mean distance to centroid (CD) and the mean nearest-neighbour distance (NND) are given. Information on the trophic length of the community is given as the δ^15^N range (NR), and an estimate of the diversity of basal resources is provided by the δ^13^C range (CR).

## Results

**Fig. 2:**
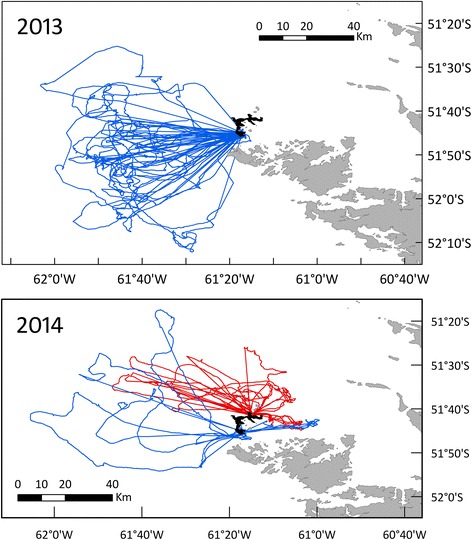
First foraging trips for Gentoo Penguins *Pygoscelis papua.* The data correspond to penguins breeding at the North End (*red lines*) and South End (*blue*) colonies, New Island (in *black*), Falkland Islands / Islas Malvinas, during chick guard (December) in 2013 and 2014. Only the first foraging trip of each individual was included

**Fig. 3:**
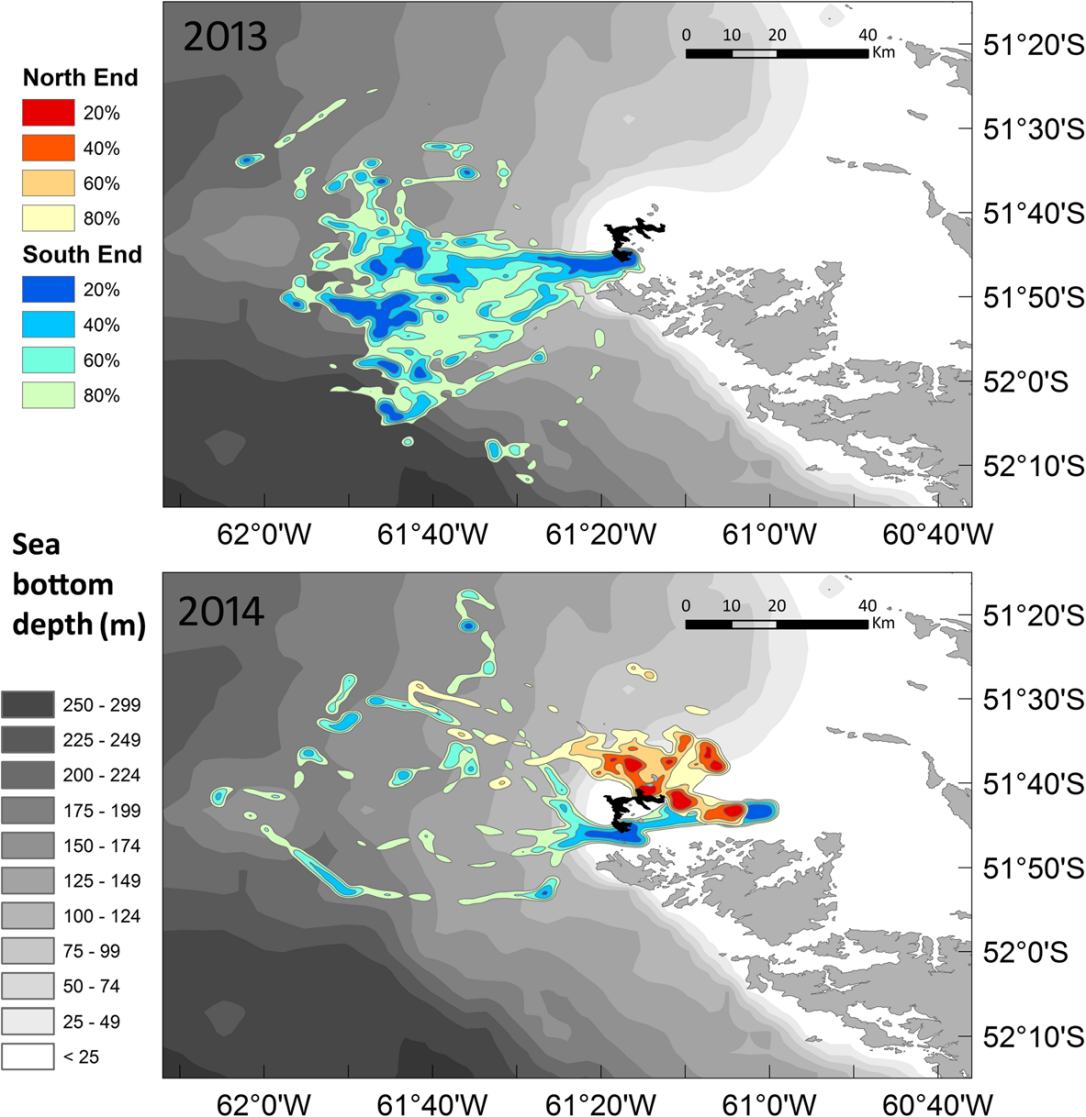
Kernel density distribution (20, 40, 60, and 80%) of dive locations. Kernel density distribution shows the places where the Gentoo Penguins *Pygoscelis papua* spent most of their forging time, for birds breeding at the North End (*shades of red*) and South End (*shades of blue*) colonies, New Island (in *black*), Falkland Islands / Islas Malvinas. Depth zones (in m) are based on data from the Global Topography (Smith & Sandwell 1997) and an IDW interpolation in ArcGIS 9.3. Only dives performed during the first foraging trip of each individual were included

The marine areas used by Gentoo Penguins varied among years, and so did the degree of spatial segregation between colonies (Fig. 2). This was most evident when kernel densities were considered (Fig. 3). In 2013, birds from the South End colony performed the longest trips, which took them furthest away from the colonies and which were more extended in time (Table 1). However, most trip parameters did not differ significantly between colonies or between years due to large inter-individual variability (Table 1).

The mean number of dives per foraging trip was similar for both colonies and years (Table 2). Birds from the South End colony carried out more pelagic dives in 2013, while the proportion of pelagic and benthic dives was almost equal for both colonies in 2014 (Table 2). The maximum dive depth was achieved by a bird from the South End colony in 2013 (Table 2). However, the median of the event maximum depth was largest in 2014 and showed no differences between colonies (Table 2). The deepest pelagic dives corresponded to birds from the North End colony (Table 2).

**Fig. 4:**
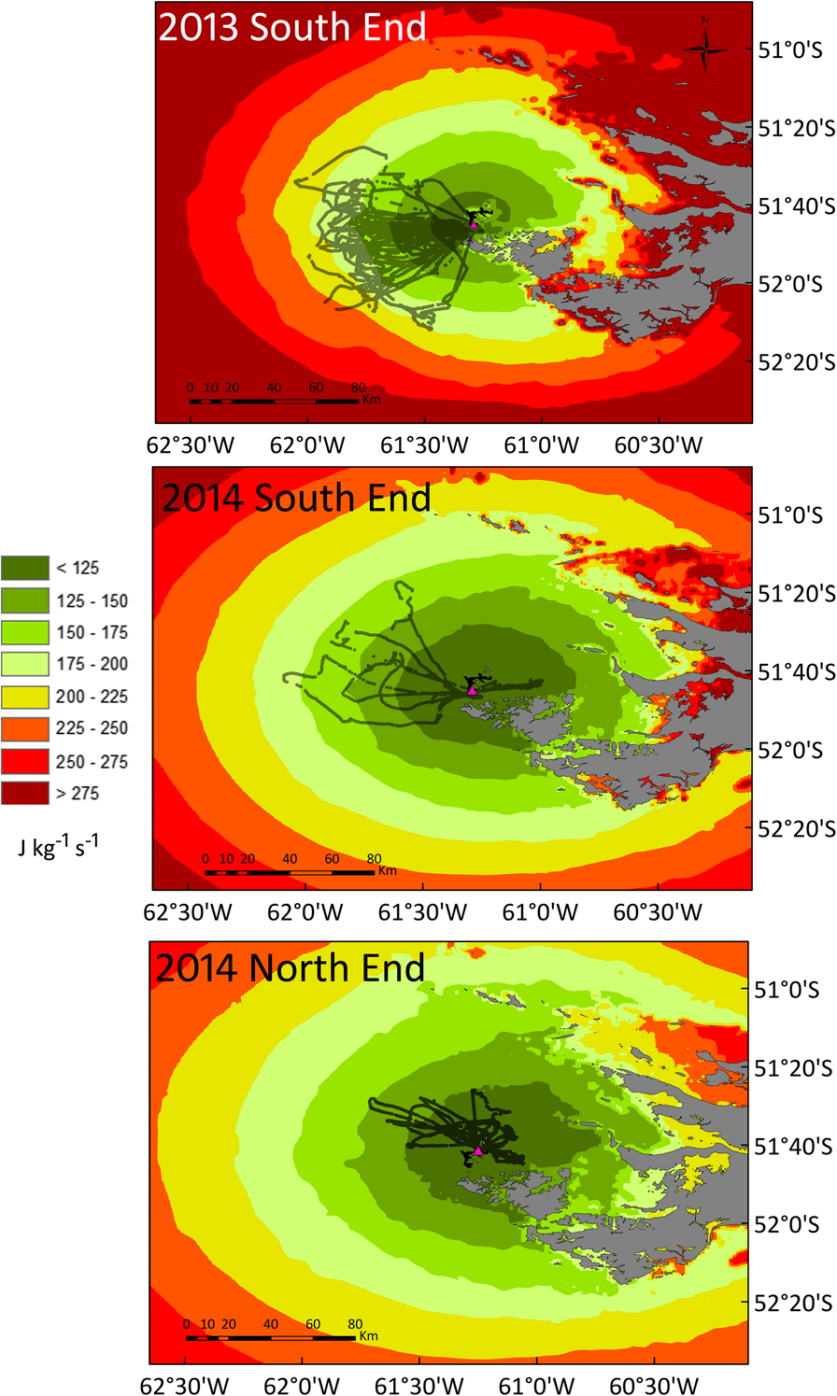
Variable energy landscapes. Energy landscapes based on the bathymetry around New Island and the mass-specific total cost of foraging (diving plus commuting) by Gentoo Penguins *Pygoscelis papua* relative to the bottom time (in J kg^−1^ s^−1^), taking into account the different proportion of benthic and pelagic dives carried out by the penguins in each colony and breeding season

Gentoo Penguins preferentially used the areas of the energy landscape that resulted in lower foraging costs per bottom time gain, mostly below 225 J kg^−1^ s^−1^ in 2013 and below 175 J kg^−1^ s^−1^ in 2014 (Fig. 4). There was no evident relationship between the foraging areas used by the Gentoo Penguins and depth or distance to the colony (Fig. 3). The selection of the foraging areas varied noticeably in space (Figs. 2 and 3) and water depth (Fig. 3, Additional file 1: Figure S1), but in all cases implied minimal power requirements compared with other parts of the landscape accessible to the penguins around the colony (Fig. 4).

**Fig. 5:**
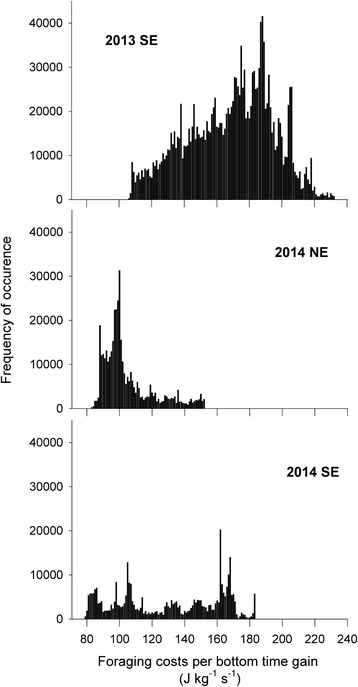
Frequencies of foraging costs per bottom time gain. Data are shown in J kg^−1^ s^−1^, for each colony and breeding season of Gentoo Penguins *Pygoscelis papua* breeding at the North End and South End colonies, New Island, Falkland Islands / Islas Malvinas, during chick guard (December) in 2013 and 2014

The energy landscapes varied strongly in time (i.e. between the 2 years), but no obvious differences were observed between the energy landscapes calculated for the two colonies in 2014 (Fig. 4). We compared the foraging costs per bottom time gain extracted from the energy landscapes and corresponding to the locations where actual dive events were carried out (distribution pattern shown in Fig. 5). When comparing the means for each deployed penguin, the highest mean foraging costs per bottom time gain was observed for the South End colony in 2013 (mean ± SD, 2013: 163.7 ± 9.7, 2014: 107.8 ± 22.2, J kg^−1^ s^−1^; *t* = 7.790, d.f. = 17, *P* < 0.001). No differences in foraging costs per bottom time gain were observed between the colonies in 2014 (South End: 107.8 ± 22.2, North End: 106.7 ± 13.8, J kg^−1^ s^−1^; *t* = 0.109, d.f. = 11, *P* = 0.915).

At the beginning of the fieldwork (December, i.e. late incubation and early chick-feeding), we counted all active nests at the colonies. The North End colony consisted of 2378 nests in 2013 and 2073 nests in 2014. The South End colony contained 2044 nests in 2013 and 2072 nests in 2014. During the crèche period (mid-January), the colonies were revisited to count the number of chicks as a measure of breeding success. The North End colony contained 1352 chicks in 2013 and 3172 in 2014. In the South End colony we counted 2458 chicks in 2013 and 2171 chicks in 2014. However, the South End colony was affected by an outbreak of avian pox in January 2015, which affected the numbers corresponding to the second season of this study (December 2014 to February 2015). Despite this disease, the overall breeding success was higher in 2014 (1.29 chick per nest) than in 2013 (0.86 chicks per nest).

### Stable isotope niche analysis

**Fig. 6:**
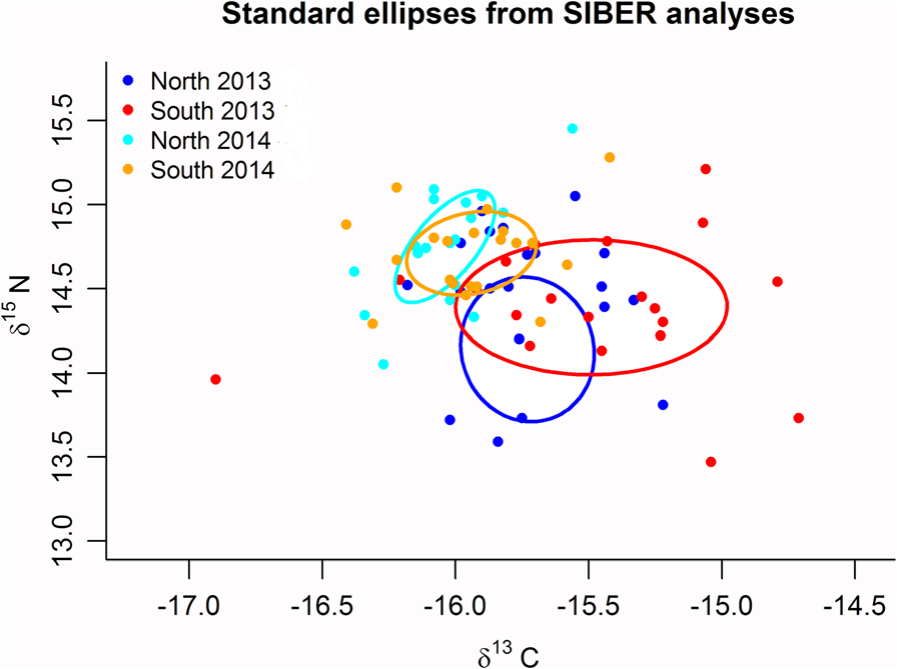
Isotopic niches based on δ^13^C and δ^15^N. Values were measured in feathers from Gentoo Penguin *Pygoscelis papua* chicks grown at the North End and South End colonies, New Island, during the breeding seasons 2013 and 2014

The SIBER analyses corresponding to Gentoo Penguin chick feathers revealed differences between the years (Fig. 6, Table 3). In 2014, we measured lower δ^13^C (GLM, effect of site: *F* = 5.66, *P* = 0.020, effect of year: *F* = 26.68, *P* < 0.001) and higher δ^15^N isotope values (GLM, effect of site: *F* = 0.37, *P* = 0.544, effect of year: *F* = 14.92, *P* < 0.001). All niche metrics (Table 3) were larger in 2013 than in 2014, indicating a higher variability in the feeding ecology among individuals. Furthermore, the South End colony (which was represented by the birds carrying data loggers) had the highest niche metrics among all four groups (Table 3).

**Table 3:** Isotopic niche metrics of Gentoo Penguins *Pygoscelis papua.* Parameters are based on carbon (δ^13^C) and nitrogen (δ^15^N) stable isotopes of chick feather samples as a marker of breeding season foraging ecology from two colonies at New Island and two breeding seasons calculated with the SIAR package. *SE* South End colony, *NE* North End colony

Symbol	Explanation	NE, 2013	SE, 2013	NE, 2014	SE, 2014
		*n* = 20	*n* = 20	*n* = 18	*n* = 20
LOC	Location of centroid (mean δ^13^C, mean δ^15^N)	−15.73, 14.45	−15.49, 14.39	−16.04, 14.75	−15.94, 14.71
SEA	Area of the standard ellipse (isotope niche width)	0.34	0.63	0.15	0.19
SEAc	as above, corrected for sample size	0.36	0.67	0.16	0.20
NR	trophic length (range in δ^15^N)	1.46	1.74	1.40	0.99
CR	diversity of basal resources (range in δ^13^C)	0.96	2.19	0.82	0.99
CD	niche width 2 (Mean distance to centroid)	0.43	0.52	0.31	0.29
NND	mean Nearest Neighbour Distance	0.16	0.26	0.13	0.15

## Discussion

The costs associated to movements are frequently determined by the landscapes through which animals move [7, 12, 21]. Hence, the energy landscape approach to movement ecology predicts that individuals will modulate different foraging parameters in order to maximize net energy gain during foraging avoiding costly areas [21, 22, 24].

As in previous studies of animal movement, Gentoo penguins in our study consistently foraged in areas of the energy landscape that resulted in lower foraging costs. However, the results of the present study show that, in line with our prediction, the energy landscape changed temporally, namely between the two seasons. During the first season, in December 2013, Gentoo penguins experienced an energy landscape with increased foraging costs around New Island when compared to the second season, in December 2014. Despite these higher costs, Gentoo Penguins in 2013 travelled further (albeit not statistically significantly), and foraged most of the time in more costly areas of the energy landscape than in 2014 (Fig. 5). The breeding success data were in line with this: in a situation of higher energy expenditure (2013), the breeding success was low (0.86 chicks/nest), compared to a situation of lower energy expenditure (2014: 1.29 chicks/nest).

Variation in energy landscapes over time may be due to changes in the landscapes that make the movements of the animal more challenging [21]. In marine environments or “seascapes”, the energy landscapes may vary in time following changing oceanographic conditions or as a consequence of fluctuating food availability. In the Falkland Islands, the total catches of rock cod and Patagonian longfin squid, the two main items in the diet of Gentoo Penguin during guard [40], were lower in 2013 (32,436 and 40,168 t respectively) than in 2014 (56,686 and 48,702 t respectively) [71]. The Falkland Islands fisheries statistics thus suggested lower food availability during 2013 compared with 2014 [71], which was reflected in the more expensive energy landscape. This is also in agreement with the lower chlorophyll *a* concentrations observed in the area southwest of the Falkland Islands in 2013 (see Additional file 1: Figure S17, A) with respect to 2014 (see Additional file 1: Figure S17, B; Giovanni Ocean Color Time-Series, National Aeronautics and Space Administration, USA). Also during 2013, we observed a predominance of lobster krill remains in the scats of Gentoo Penguins breeding on New Island. Previous studies showed that lobster krill is a less preferred item in the diet of Gentoo Penguins at the Falkland Islands both during guard and crèche formation [39, 40]. The reduced availability of preferred prey and the generally lower ocean productivity may have forced the Gentoo Penguins from New Island to prey on a less preferred prey in 2013. Additionally, δ^15^N was lower in 2013, suggesting lower trophic level prey (e.g. lobster krill), and all δ^13^C and δ^15^N niche metrics were larger in 2013 than in 2014 (Table 3), suggesting a higher variability in the feeding ecology among individuals.

A high degree of plasticity in foraging behaviour and diet was also reported for Gentoo Penguins both from Antarctica and Sub-Antarctic islands as a buffer against changes in prey availability [29, 35, 72]. Similarly, in our study of Imperial Shags at New Island, we also observed such plasticity in the diet, diving, and foraging behaviour over time [16]. In the case of Imperial Shags, pelagic dives dominated in poorer years in terms of breeding success. In our present study, Gentoo Penguins performed a significantly higher proportion of pelagic dives during 2013 (Table 2), probably preying on the pelagic phase of lobster krill [73]. This switch to a predominantly pelagic foraging strategy in 2013 could be interpreted as a strategy to overcome a more expensive energy landscape. In years when food availability makes benthic foraging altogether too costly, birds could switch to a more cost-effective pelagic strategy.

The balance between energy gain and variable energy costs of foraging will directly affect the survival and reproduction of individuals in a particular landscape [22, 24]. It follows that in the context of natural selection individuals that move efficiently to areas of the best energy gain per energy expenditure will increase their fitness, leading to the evolution of a variety of energy-saving mechanisms [22]. However, this could be a too simplistic approach, as movement can also depend on other factors in addition to the availability of prey, like the probability of being predated [11, 12, 21, 74].

Gentoo Penguins from New Island did not forage in all areas of the energy landscape with lower foraging costs. An area with the lowest foraging costs i.e. < 125 J kg^−1^ s^−1^ located to the north-west of New Island was avoided in both years of this study and also during a previous study (Figs. 2, 3 and 4) [5]. This area surrounds three South American Fur Seal *Arctocephalus australis* colonies (Fig. 1). According to the landscape of fear approach to movement ecology [12, 75], the spatial and temporal use of the landscapes would be driven by the fear of being killed (risk of predation). Our results are also in line with the landscape of fear approach, adding to a number of studies showing the importance of non-lethal effects of predation on seabird foraging behaviour (e.g. [11]). Moreover, the foraging movements observed during this study provide further support to the complementarity of the energy and fear landscape paradigms proposed by Gallagher et al. [12], as a way of better understanding the mechanistic basis of movement ecology.

## Conclusions

This study clearly illustrates that in order to adequately understand the mechanistic basis of movement ecology it is necessary to consider a variety of factors and complementary approaches. A complementary approach looking at the energy gain and variable energy costs of foraging (energy landscapes) and the non-lethal effects of predation (landscape of fear) that also considers the fluctuations in food availability and/or the spatial and temporal changes of the landscapes will certainly help us understanding the complex decisions made by wild animals during foraging. Energy landscapes are also useful in linking energy gain and variable energy costs of foraging to breeding success. Thus, long term studies of the energy landscapes experienced by populations of wild animals could also help understanding demographic changes and their consequences for conservation. Moreover, investigating energy landscapes over time may become a useful tool for the identification of key areas for conservation spatial planning.

## Additional file

Additional file 1:
**Table S1**. GAM investigating the sum of ODBAas a function of maximum dive depth. **Table S2**. Relationship between the sum of ODBA and maximum dive depth. **Table S3**. GAM investigating the bottom time as a function of event maximum depth. **Table S4**. Relationship between bottom time and event maximum depth. **Figure S1**. Depth zones. **Figure S2**. Distribution of depth during benthic (A) and pelagic (B) dives. **Figure S3**. Distribution in different depths of benthic (A) and pelagic (B) dives. **Figure S4**. Benthic dives example. **Figure S5**.Sum of ODBA versus maximum dive depth for benthic dives (South End, 2013). **Figure S6**. Sum of ODBA versus maximum dive depth for pelagic dives (South End, 2013). **Figure S7**. Sum of ODBA versus maximum dive depth for benthic dives (South End, 2014). **Figure S8**. Sum of ODBA versus maximum dive depth for pelagic dives (South End, 2014). **Figure S9**. Sum of ODBA versus maximum dive depth for benthic dives (North End, 2014). **Figure S10**. Sum of ODBA versus maximum dive depth for pelagic dives (North End, 2014). **Figure S11**. Bottom time versus event maximum depth for benthic dives (South End, 2013). **Figure S12**. Bottom time versus event maximum depth for pelagic dives (South End, 2013). **Figure S13**. Bottom time versus event maximum depth for benthic dives (South End, 2014). **Figure S14**. Bottom time versus event maximum depth for pelagic dives (South End, 2014). **Figure S15**. Bottom time versus event maximum depth for benthic dives (North End, 2014). **Figure S16**. Bottom time versus event maximum depth for pelagic dives (North End, 2014). **Figure S17**. Chlorophyll a concentration. (DOCX 2492 kb)

## Abbreviations

Axy: Micro tri-axial accelerometer
BBT: Benthic bottom time
CD: Distance to centroid
CF-IRMS: Continuous-flow isotope ratio mass spectrometry
CR: Carbon range
CT: Cost of travelling
DD: Dive duration
GAM: Generalized additive models
GLM: General linear model
GPS-TD logger: General position system-temperature-depth logger
IDW: Inverse distance weighted
LOC: Location of centroid
mBBT: Minimum benthic bottom times
MND: Mean number of dives per foraging trip
MP: Mass-specific power
NND: Nearest-neighbour distance
NR: Nitrogen range
ODBA: Overall dynamic body acceleration
pBD: Proportion of benthic dives
PBT: Pelagic bottom times
pPD: Proportion of pelagic dives
SEA: Standard ellipse area
SIBER: Stable isotope bayesian ellipses
TBT: Total bottom time
TCF: Total cost of foraging
TRC: Total relative cost
TT: Travel time

## References

[1] Wolf JBW, Kauermann G, Trillmich F (2005). Males in the shade: habitat use and sexual segregation in the Galapagos sea lion (*Zalophus californianus wollebaeki*). Behav Ecol Sociobiol.

[2] Nathan R, Getz WM, Revilla E, Holyoak M, Kadmon R, Saltz D, Smouse PE (2008). A movement ecology paradigm for unifying organismal movement research. Proc Natl Acad Sci U S A.

[3] Revilla E, Wiegand T (2008). Movement ecology special feature: individual movement behavior, matrix heterogeneity, and the dynamics of spatially structured populations. Proc Natl Acad Sci U S A.

[4] Ballard G, Dugger KM, Nur N, Ainley DG (2010). Foraging strategies of Adelie penguins: adjusting body condition to cope with environmental variability. Mar Ecol Prog Ser.

[5] Masello JF, Mundry R, Poisbleau M, Demongin L, Voigt CC, Wikelski M, Quillfeldt P (2010). Diving seabirds share foraging space and time within and among species. Ecosphere.

[6] Wilson RP (2010). Resource partitioning and niche hyper-volume overlap in free-living Pygoscelid penguins. Funct Ecol.

[7] Wall J, Douglas-Hamilton I, Vollrath F (2006). Elephants avoid costly mountaineering. Curr Biol.

[8] Wilcove DS, Wikelski M (2008). Going, Going, Gone: Is Animal Migration Disappearing?. PLoS Biol.

[9] MacArthur RH, Pianka ER (1966). On the optimal use of a patchy environment. Am Nat.

[10] Schoener TW (1971). Theory of feeding strategies. Annu Rev Ecol Syst.

[11] Riou S, Hamer KC (2008). Predation risk and reproductive effort: impacts of moonlight on food provisioning and chick growth in Manx shearwaters. Anim Behav.

[12] Gallagher AJ, Creel S, Wilson RP, Cooke SJ (2017). Energy landscapes and the landscape of fear. Trends Ecol Evol.

[13] Holyoak M, Casagrandi R, Nathan R, Revilla E, Spiegel O (2008). Trends and missing parts in the study of movement ecology. Proc Natl Acad Sci U S A.

[14] Holland RA, Wikelski M, Kümmeth F, Bosque C (2009). The secret life of oilbirds: new insights into the movement ecology of a unique avian frugivore. PLoS One.

[15] Roshier DA, Doerr VAJ, Doerr ED (2008). Animal movement in dynamic landscapes: interaction between behavioural strategies and resource distributions. Oecologia.

[16] Quillfeldt P, Schroff S, van Noordwijk HJ, Michalik A, Ludynia K, Masello JF (2011). Flexible foraging behavior of a sexually dimorphic seabird: large males do not always dive deep. Mar Ecol Prog Ser.

[17] Langman VA, Roberts TJ, Black J, Maloiy GMO, Heglund NC, Weber J-M, Kram R, Taylor CR (1995). Moving cheaply: energetics of walking in the African elephant. J Exp Biol.

[18] Elliott KH, Davoren GK, Gaston AJ (2008). Increasing energy expenditure for a deep-diving bird alters time allocation during the dive cycle. Anim Behav.

[19] Ballance LT, Ainley DG, Ballard G, Barton K (2009). An energetic correlate between colony size and foraging effort in seabirds, an example of the Adelie penguin *Pygoscelis adeliae*. J Avian Biol.

[20] Rubenson J, Henry HT, Dimoulas PM, Marsh RL (2006). The cost of running uphill: linking organismal and muscle energy use in guinea fowl (*Numida meleagris*). J Exp Biol.

[21] Wilson RP, Quintana F, Hobson VJ (2012). Construction of energy landscapes can clarify the movement and distribution of foraging animals. Proc R Soc B.

[22] Shepard ELC, Wilson RP, Rees WG, Grundy E, Lambertucci SA, Simon BV (2013). Energy landscapes shape animal movement ecology. Am Nat.

[23] Brownscombe JW, Gutowsky LF, Danylchuk AJ, Cooke SJ (2014). Foraging behaviour and activity of a marine benthivorous fish estimated using tri-axial accelerometer biologgers. Mar Ecol Prog Ser.

[24] Mosser AA, Avgar T, Brown GS, Walker CS, Fryxell JM (2014). Towards an energetic landscape: broad-scale accelerometry in woodland caribou. J Anim Ecol.

[25] Boersma PD (1978). Breeding patterns of Galapagos penguins as an indicator of oceanographic conditions. Science.

[26] Ballance LT, Pitman RL, Fiedler PC (2006). Oceanographic influences on seabirds and cetaceans of the eastern tropical Pacific: a review. Prog Oceanogr.

[27] Quillfeldt P, Strange I, Masello JF (2007). Sea surface temperatures, variable food supply and behavioural buffering capacity in thin-billed prions *Pachyptila belcheri*: breeding success, provisioning and chick begging. J Avian Biol.

[28] Quillfeldt P, Masello JF, McGill RAR, Adams M, Furness RW (2010). Moving polewards in winter: a recent change in migratory strategy. Front Zool.

[29] Miller AK, Karnovsky NJ, Trivelpiece WZ (2009). Flexible foraging strategies of gentoo penguins *Pygoscelis papua* over 5 years in the south Shetland Islands, Antarctica. Mar Biol.

[30] Fort J, Cherel Y, Harding AMA, Welcker J, Jakubas D, Steen H, Karnovsky NJ, Gremillet D (2010). Geographic and seasonal variability in the isotopic niche of little auks. Mar Ecol Prog Ser.

[31] Shealer DA, Schreiber EA, Burger J (2002). Foraging behavior and food of seabirds. Biology of marine birds.

[32] Lescroël A, Bost C-A (2005). Foraging under contrasting oceanographic conditions: the gentoo penguin at Kerguelen archipelago. Mar Ecol Prog Ser.

[33] Aronson RB, Givnish TJ (1983). Optimal central-place foragers: a comparison with null hypotheses. Ecology.

[34] Ropert-Coudert Y, Gremillet D, Kato A, Ryan PG, Naito Y, Le Maho Y (2004). A fine-scale time budget of cape gannets provides insights into the foraging strategies of coastal seabirds. Anim Behav.

[35] Hinke JT, Salwicka K, Trivelpiece SG, Watters GM, Trivelpiece WZ (2007). Divergent responses of Pygoscelis penguins reveal a common environmental driver. Oecologia.

[36] Agnew DJ (2002). Critical aspects of the Falkland Islands pelagic ecosystem: distribution, spawning and migration of pelagic animals in relation to oil exploration. Aquat Conserv Mar Freshwat Ecosyst.

[37] Arkhipkin A, Brickle P, Laptikhovsky V (2010). The use of island water dynamics by spawning red cod, *Salilota australis* (Pisces: Moridae) on the Patagonian shelf (Southwest Atlantic). Fish Res.

[38] Strange I, Catry P, Strange G, Quillfeldt P (2007). New Island, Falkland Islands. A South Atlantic wildlife sanctuary for conservation management.

[39] Clausen AP, Pütz K (2002). Recent trends in diet composition and productivity of gentoo, magellanic and rockhopper penguins in the Falkland Islands. Aquat Conserv Mar Freshwat Ecosyst.

[40] Handley JM, Baylis AM, Brickle P, Pistorius P (2016). Temporal variation in the diet of gentoo penguins at the Falkland Islands. Polar Biol.

[41] Thompson KR: An assessment of the potential for competition between seabirds and fisheries in the Falkland Islands**.** In Falkland Islands Foundation project report. Brighton: Falkland Islands Foundation; 1989.

[42] Thompson KR (1994). Predation on *Gonatus antarcticus* by Falkland Islands seabirds. Antarct Sci.

[43] Clausen AP, Arkhipkin AI, Laptikhovsky VV, Huin N (2005). What is out there: diversity in feeding of gentoo penguins (*Pygoscelis papua*) around the Falkland Islands (Southwest Atlantic). Polar Biol.

[44] Pütz K, Ingham RJ, Smith JG (2002). Foraging movements of magellanic penguins *Spheniscus magellanicus* during the breeding season in the Falkland Islands. Aquat Conserv Mar Freshwat Ecosyst.

[45] Wilson RP, Pütz K, Peters G, Culik B, Scolaro JA, Charassin J-B, Ropert-Coudert Y (1997). Long-term attachment of transmitting and recording devices to penguins and other seabirds. Wildl Soc Bull.

[46] Ludynia K, Dehnhard N, Poisbleau M, Demongin L, Masello JF, Quillfeldt P (2012). Evaluating the impact of handling and logger attachment on foraging parameters and physiology in southern Rockhopper penguins. PLoS One.

[47] Grémillet D, Dell'Omo G, Ryan PG, Peters G, Ropert-Coudert Y, Weeks SJ (2004). Offshore diplomacy, or how seabirds mitigate intra-specific competition: a case study based on GPS tracking of cape gannets from neighbouring colonies. Mar Ecol Prog Ser.

[48] Garthe S, Montevecchi WA, Chapdelaine G, Rail JF, Hedd A (2007). Contrasting foraging tactics by northern gannets (*Sula bassana*) breeding in different oceanographic domains with different prey fields. Mar Biol.

[49] Garthe S, Montevecchi WA, Davoren GK (2007). Flight destinations and foraging behaviour of northern gannets (*Sula bassana*) preying on a small forage fish in a low-Arctic ecosystem. Deep-Sea Research II.

[50] Mattern T, Ellenberg U, Houston DM, Davis LS (2007). Consistent foraging routes and benthic foraging behaviour in yellow-eyed penguins. Mar Ecol Prog Ser.

[51] Wilson RP, Shepard ELC, Liebsch N (2008). Prying into the intimate details of animal lives: use of a daily diary on animals. Endanger Species Res.

[52] Smith WH, Sandwell DT (1997). Global sea floor topography from satellite altimetry and ship depth soundings. Science.

[53] Global Topography Scripps Institution of Oceanography, University of California San Diego, La Jolla, USA. 2017. http://topex.ucsd.edu/WWW_html/mar_topo.html. Accessed 3 July 2017.

[54] Tremblay Y, Cherel Y (2000). Benthic and pelagic dives: a new foraging behaviour in rockhopper penguins. Mar Ecol ProgSer.

[55] Tremblay Y, Cook TR, Cherel Y (2005). Time budget and diving behaviour of chick-rearing Crozet shags. Can J Zool.

[56] Wood AG, Naef-Daenzer B, Prince PA, Croxall JP (2000). Quantifying habitat use in satellite-tracked pelagic seabirds: application of kernel estimation to albatross location. J Avian Biol.

[57] Beyer HL: Hawth's analysis Tools for ArcGIS**.** 2004. http://www.spatialecology.com/htools. Accessed 3 July 2017.

[58] R Development Core Team (2016). R: a language and environment for statistical computing.

[59] Wilson RP, White CR, Quintana F, Halsey LG, Liebsch N, Martin GR, Butler PJ (2006). Moving towards acceleration for estimates of activity-specific metabolic rate in free-living animals: the case of the cormorant. J Anim Ecol.

[60] Halsey LG, Green JA, Wilson RP, Frappell PB (2009). Accelerometry to estimate energy expenditure during activity: best practice with data loggers. Physiol Biochem Zool.

[61] Halsey LG, Shepard ELC, Quintana F, Gómez Laich A, Green JA, Wilson RP (2009). The relationship between oxygen consumption and body acceleration in a range of species. Comparative Biochemistry and Physiology A.

[62] Shepard ELC, Wilson RP, Quintana F, Gómez Laich A, Forman DW (2009). Pushed for time or saving on fuel: fine-scale energy budgets shed light on currencies in a diving bird. Proc R Soc B.

[63] Gleiss AC, Wilson RP, Shepard EL (2011). Making overall dynamic body acceleration work: on the theory of acceleration as a proxy for energy expenditure. Methods Ecol Evol.

[64] Halsey LG (2017). Relationships grow with time: a note of caution about energy expenditure-proxy correlations, focussing on accelerometry as an example. Funct Ecol.

[65] Culik B, Wilson R, Dannfeld R, Adelung D, Spairani H, Coria NRC (1991). Pygoscelid penguins in a swim canal. Polar Biol.

[66] Elliott KH (2016). Measurement of flying and diving metabolic rate in wild animals: review and recommendations. Comp Biochem Physiol A Mol Integr Physiol.

[67] Heldmaier G, Neuweiler G, Rössler W (2013). Vergleichende Tierphysiologie.

[68] Johnston K, Ver Hoef JM, Krivoruchko K, Lucas N: Using ArcGIS geostatistical analyst. Redlands: ESRI; 2001.

[69] Jackson AL, Inger R, Parnell AC, Bearhop S (2011). Comparing isotopic niche widths among and within communities: SIBER – stable isotope Bayesian ellipses in R. J Anim Ecol.

[70] Layman CA, Arrington DA, Montaña CG, Post DM (2007). Can stable isotope ratios provide for community-wide measures of trophic structure?. Ecology.

[71] Government FI: Fisheries department fisheries statistics, volume 20**,** 2015**.** pp. 1–94. Stanley: FIG Fisheries Department; 2016:1-94.

[72] Carpenter-Kling T, Handley JM, Green DB, Reisinger RR, Makhado AB, Crawford RJM, Pistorius PA (2017). A novel foraging strategy in gentoo penguins breeding at sub-Antarctic Marion Island. Mar Biol.

[73] Williams BG (1980). The pelagic and benthic phases of post-metamorphic *Munida gregaria* (Fabricius) (Decapoda, Anomura). J Exp Mar Biol Ecol.

[74] Avgar T, Mosser A, Brown GS, Fryxell JM (2013). Environmental and individual drivers of animal movement patterns across a wide geographical gradient. J Anim Ecol.

[75] Laundré JW, Hernández L, Altendorf KB (2001). Wolves, elk, and bison: reestablishing the “landscape of fear” in Yellowstone National Park, USA. Can J Zool.

