# Supplementary material for: How animals distribute themselves in space: variable energy landscapes
Source: Front Zool. 2017 Jul 5;14:33. doi: 10.1186/s12983-017-0219-8 (PMC5499017; doi:10.1186/s12983-017-0219-8)

**Additional File**

**How animals distribute themselves in space: changing energy landscapes**

Juan F Masello^1,*^, Akiko Kato^2^, Julia Sommerfeld^1^, Thomas Mattern^1^, Martin Wikelski^3^ and Petra Quillfeldt^1^

^1^ Justus Liebig University Giessen, Department of Animal Ecology & Systematics, Heinrich-Buff-Ring 38, D-35392 Giessen, Germany;

^2^ Centre d’Etudes Biologiques de Chizé, UMR7372 CNRS-Université La Rochelle, 79360 Villiers en Bois, France;

^3^ Max Planck Institute for Ornithology, Vogelwarte Radolfzell, Schlossallee 2, D-78315, Radolfzell, Germany

* Correspondence: juan.f.masello@bio.uni-giessen.de

**Table S1**. Results of a General Additive Model (GAM) investigating the sum of Overall Dynamic Body Acceleration (ODBA) during dive as a function of maximum dive depth (*n* = 11284) in Gentoo Penguin *Pygoscelis papua* with the intra-depth zone (IDZ; foraging dives performed by the individuals split in benthic and pelagic), colonies at New Island (North and South End), Falkland/Malvinas Islands, and years (2013 and 2014) as factors

|  | d.f. | *F* | *P* |
| --- | --- | --- | --- |
| dive depth | 1 | 43994.1 | < 0.001 |
| year | 1 | 141.3 | < 0.001 |
| colony | 1 | 311.1 | < 0.001 |
| IDZ | 1 | 434.8 | < 0.001 |

**Table S2**. The relationship between the sum of Overall Dynamic Body Acceleration (ODBA) during dive and maximum dive depth for the different combination of Gentoo Penguin *Pygoscelis papua* colonies at New Island (North End and South End), Falkland/Malvinas Islands, study years, and between benthic and pelagic dives (based on the index of benthic diving behaviour, intra-depth zone; IDZ)

| **Year** | **Colony** | **IDZ** | **regression** | ***R^2^*** | **Description** | Paramenters | | | | |
| --- | --- | --- | --- | --- | --- | --- | --- | --- | --- | --- |
|  |  |  |  |  |  | **A** | **B** | **c** | **d** | **y0** |
| 2013 | South | Benthic | f=a*x/(b+x)+c*x | 0.90 | Hyperbola, Single Rectangular II, 3 Parameter | 37.9 | 23.9 | 0.2 |  |  |
| 2013 | South | Pelagic | f=a*x/(b+x)+c*x | 0.89 | Hyperbola, Single Rectangular II, 3 Parameter | 40.1 | 25.3 | 0.1 |  |  |
| 2014 | South | Benthic | f=y0+a*(1-exp(-b*x))+c*(1-exp(-d*x)) | 0.86 | Exponential Rise to Maximum, Double, 5 Parameter | 11.3 | 0.1 | 52.0 | 0.02 | -1.9 |
| 2014 | South | Pelagic | f=y0+a*(1-exp(-b*x))+c*(1-exp(-d*x)) | 0.89 | Exponential Rise to Maximum, Double, 5 Parameter | 54.9 | 0.01 | 18.8 | 0.1 | -1.4 |
| 2014 | North | Benthic | f=y0+a*(1-b^x) | 0.82 | Exponential Rise to Maximum, Simple Exponent, 3 Parameter | 68.8 | 1.0 |  |  | 3.9 |
| 2014/15 | North | Pelagic | f=y0+a*(1-exp(-b*x))+c*(1-exp(-d*x)) | 0.86 | Exponential Rise to Maximum, Double, 5 Parameter | 59.1 | 0.01 | 13.5 | 0.2 | -3.5 |

**Table S3**. Results of a General Additive Model (GAM) investigating the bottom time as a function of event maximum depth (n = 17238; maximum depth [m] reached during dive event) in Gentoo Penguin *Pygoscelis papua* with the intra-depth zone (IDZ; foraging dives performed by the individuals split in benthic and pelagic), colonies at New Island (North and South End), Falkland/Malvinas Islands, and study years (2013 and 2014) as factors

|  | d.f. | *F* | *P* |
| --- | --- | --- | --- |
| dive depth | 1 | 12481.1 | < 0.001 |
| breeding season | 1 | 546.7 | < 0.001 |
| colony | 1 | 478.0 | < 0.001 |
| IDZ | 1 | 1706.1 | < 0.001 |

**Table S4**. The relationship between bottom time and event maximum depth for the different combination of study years, Gentoo Penguin *Pygoscelis papua* colonies at New Island (North End and South End), Falkland/Malvinas Islands, study years, and between benthic and pelagic dives (based on the index of benthic diving behaviour, intra-depth zone; IDZ)

| **BS** | **Colony** | **IDZ** | **Regression** | **R^2^** | **Description** | Paramenters | | | | | | |
| --- | --- | --- | --- | --- | --- | --- | --- | --- | --- | --- | --- | --- |
|  |  |  |  |  |  | **a** | **b** | **c** | **d** | **e** | **x_0_** | **y_0_** |
| 2013 | South | Benthic | f= y0+a/(1+exp(-(x-x0)/b)) | 0.59 | Sigmoidal, Sigmoid, 4 Parameter | 141.1 | 16.0 |  |  |  | 34.6 | -14.3 |
| 2013 | South | Pelagic | f=y0+a*x^b/(c^b+x^b) | 0.60 | Sigmoidal, Hill, 4 Parameter | 112.0 | 3.0 | 36.9 |  |  |  | 6.7 |
| 2014 | South | Benthic | f=y0+a*x^b/(c^b+x^b) | 0.43 | Sigmoidal, Hill, 4 Parameter | 76.2 | 3.1 | 27.1 |  |  |  | 18.4 |
| 2014 | South | Pelagic | f=y0+a*(1-exp(-b*x))+c*(1-exp(-d*x)) | 0.55 | Exponential Rise to Maximum, Double, 5 Parameter | 85.3 | 0.01 | 25.2 | 0.1 |  |  | -0.6 |
| 2014 | North | Benthic | f=y0+a*x^b/(c^b+x^b) | 0.45 | Sigmoidal, Hill, 4 Parameter | 61.0 | 10.9 | 44.1 |  |  |  | 42.5 |
| 2014 | North | Pelagic | f=a*x/(b+x)+c*x/(d+x)+e*x | 0.57 | Hyperbola, Double Rectangular, 5 Parameter | 17.2 | 5.7 | 4197023.5 | 34805.3 | -119.5 |  |  |

**Figure S1**. Depth (in m) zones for the marine area around New Island (in black), Falkland/Malvinas Islands, based on data from the Global Topography (Smith & Sandwell 1997) and an IDW interpolation in ArcGIS 9.3


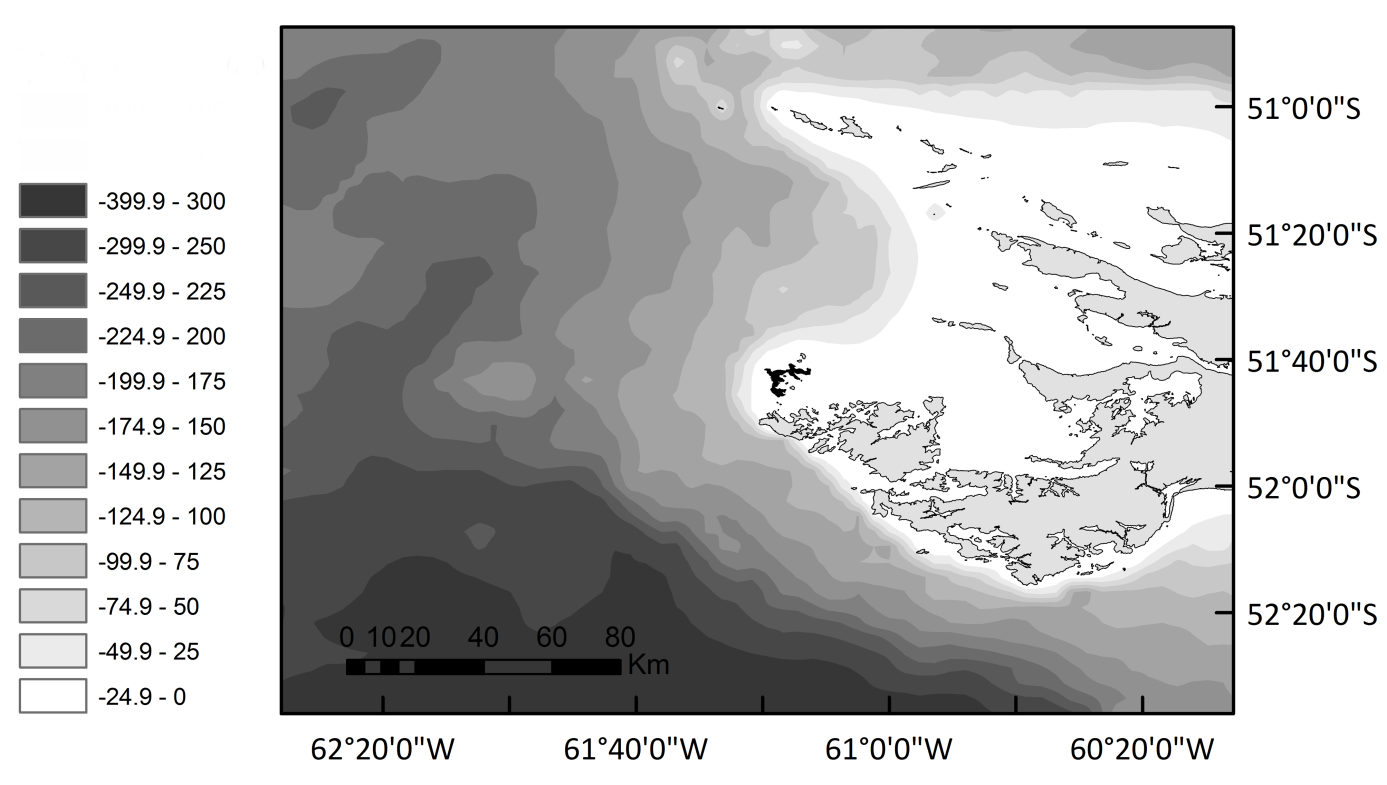


**Figure S2**. The distribution of dive depth data during benthic (A) and pelagic (B) foraging dives by Gentoo Penguin *Pygoscelis papua* breeding at New Island (Falkland/Malvinas Islands) during chick guard (December) in 2013 and 2014. Only the first foraging trip of each individual was included in order to avoid individuals with more than one trip having more weight in the data. Benthic and pelagic dives are defined with the use of the index of benthic diving behaviour, intra-depth zone (IDZ)


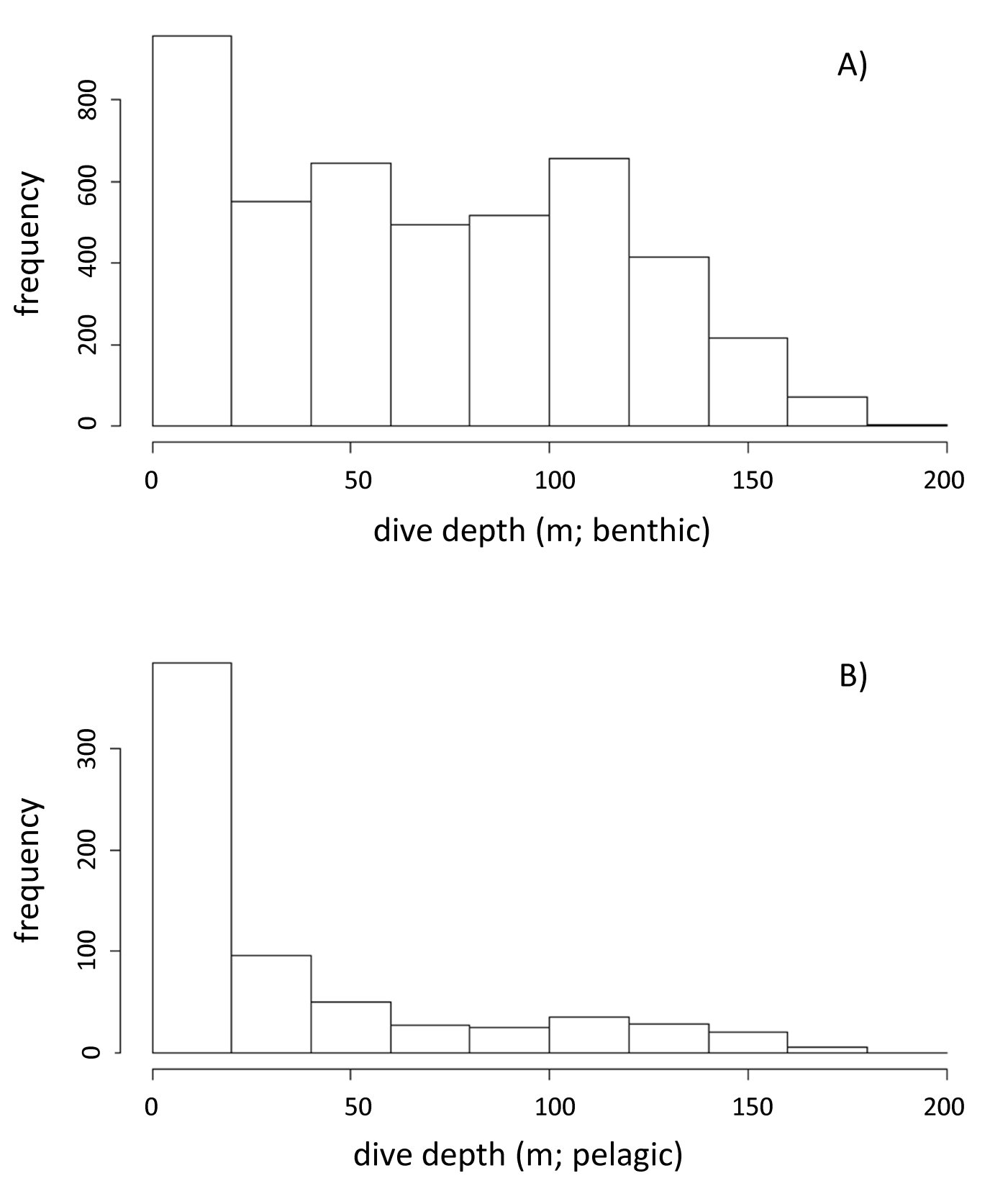


**Figure S3.** Example of the distribution in different depth zones of benthic (A) and pelagic (B) dives carried out by Gentoo Penguin *Pygoscelis papua* breeding at the South End colony (New Island, Falkland/Malvinas Islands) during chick guard (December) in 2013

(A)


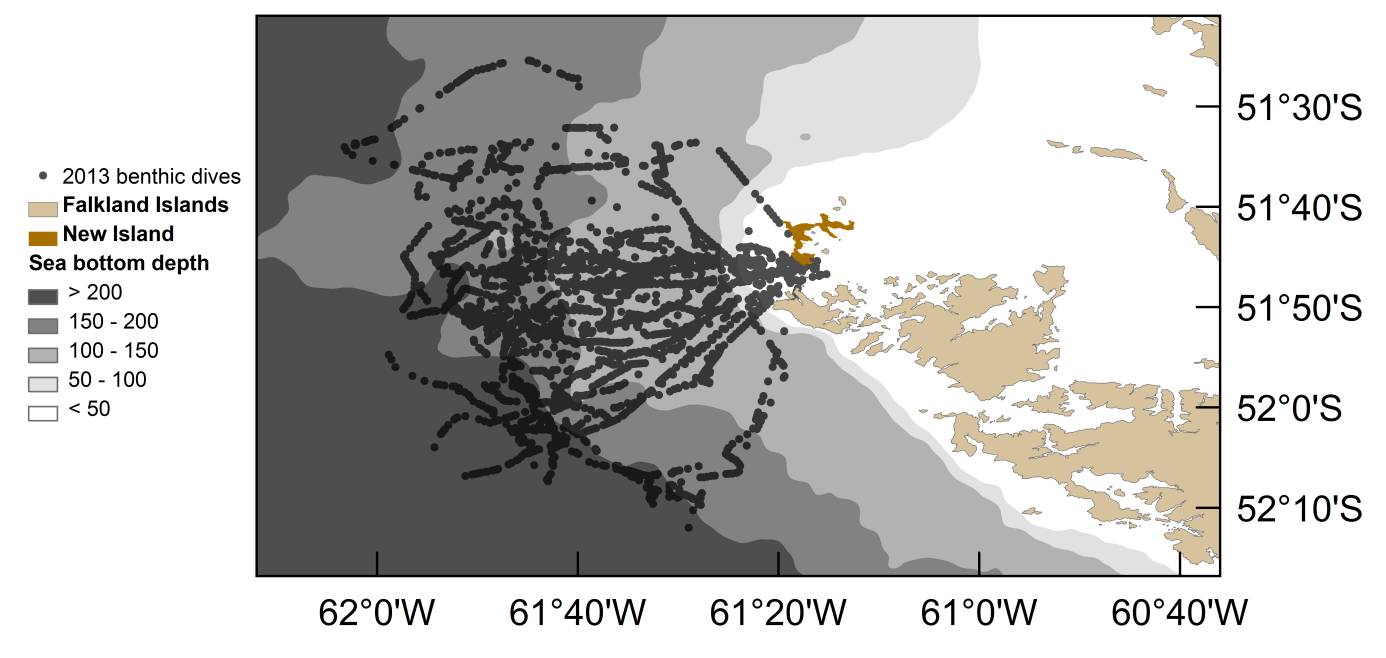


(B)


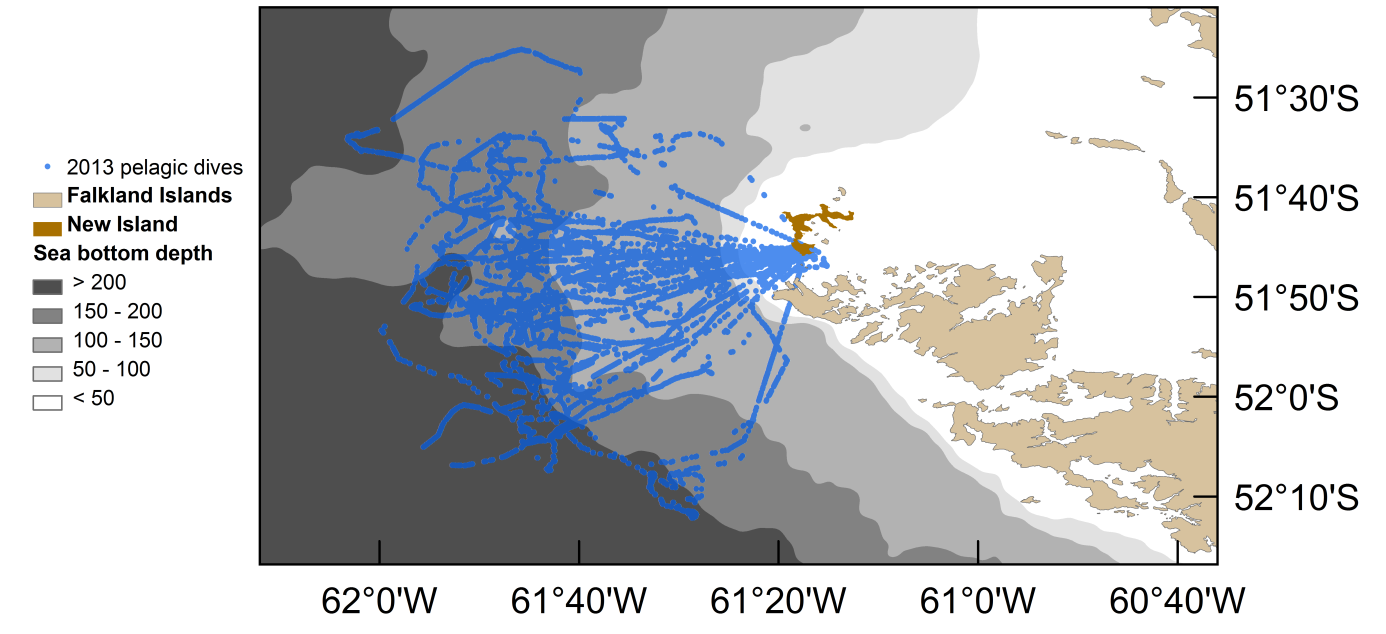


**Figure S4.** Example of benthic dives by Gentoo Penguins *Pygoscelis papua* breeding at New Island, Falkland Islands (in black). Highest water temperatures in red, lowest in green

**
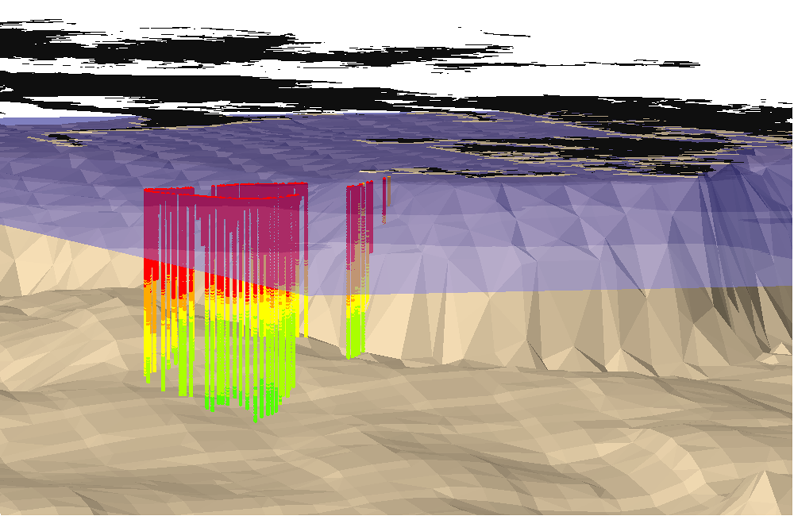
**

**Figure S5.** The relationship between the sum of Overall Dynamic Body Acceleration (ODBA) during dive and maximum dive depth for benthic dives (based on the index of benthic diving behaviour, intra-depth zone; IDZ) carried out by Gentoo Penguin *Pygoscelis papua* breeding at the South End colony (New Island, Falkland/Malvinas Islands) during chick guard (December) in 2013. Details for the regression curve are given in **Table S1**





**Figure S6.** The relationship between the sum of Overall Dynamic Body Acceleration (ODBA) during dive and maximum dive depth for pelagic dives (based on the index of benthic diving behaviour, intra-depth zone; IDZ) carried out by Gentoo Penguin *Pygoscelis papua* breeding at the South End colony (New Island, Falkland/Malvinas Islands) during chick guard (December) in 2013. Details for the regression curve are given in **Table S1**





**Figure S7.** The relationship between the sum of Overall Dynamic Body Acceleration (ODBA) during dive and maximum dive depth for benthic dives (based on the index of benthic diving behaviour, intra-depth zone; IDZ) carried out by Gentoo Penguin *Pygoscelis papua* breeding at the South End colony (New Island, Falkland/Malvinas Islands) during chick guard (December) in 2014. Details for the regression curve are given in **Table S1**





**Figure S8.** The relationship between the sum of Overall Dynamic Body Acceleration (ODBA) during dive and maximum dive depth for pelagic dives (based on the index of benthic diving behaviour, intra-depth zone; IDZ) carried out by Gentoo Penguin *Pygoscelis papua* breeding at the South End colony (New Island, Falkland/Malvinas Islands) during chick guard (December) in 2014. Details for the regression curve are given in **Table S1**





**Figure S9.** The relationship between the sum of Overall Dynamic Body Acceleration (ODBA) during dive and maximum dive depth for benthic dives (based on the index of benthic diving behaviour, intra-depth zone; IDZ) carried out by Gentoo Penguin *Pygoscelis papua* breeding at the North End colony (New Island, Falkland/Malvinas Islands) during chick guard (December) in 2014. Details for the regression curve are given in **Table S1**





**Figure S10.** The relationship between the sum of Overall Dynamic Body Acceleration (ODBA) during dive and maximum dive depth for pelagic dives (based on the index of benthic diving behaviour, intra-depth zone; IDZ) carried out by Gentoo Penguin *Pygoscelis papua* breeding at the North End colony (New Island, Falkland/Malvinas Islands) during chick guard (December) in 2014. Details for the regression curve are given in **Table S1**





**Figure S11.** The relationship between the bottom time and the event maximum depth for benthic dives (based on the index of benthic diving behaviour, intra-depth zone; IDZ) carried out by Gentoo Penguin *Pygoscelis papua* breeding at the South End colony (New Island, Falkland/Malvinas Islands) during chick guard (December) in 2013. Details for the regression curve are given in **Table S4**





**Figure S12.** The relationship between the bottom time and the event maximum depth for pelagic dives (based on the index of benthic diving behaviour, intra-depth zone; IDZ) carried out by Gentoo Penguin *Pygoscelis papua* breeding at the South End colony (New Island, Falkland/Malvinas Islands) during chick guard (December) in 2013. Details for the regression curve are given in **Table S4**





**Figure S13.** The relationship between the bottom time and the event maximum depth for benthic dives (based on the index of benthic diving behaviour, intra-depth zone; IDZ) carried out by Gentoo Penguin *Pygoscelis papua* breeding at the South End colony (New Island, Falkland/Malvinas Islands) during chick guard (December) in 2014. Details for the regression curve are given in **Table S4**





**Figure S14.** The relationship between the bottom time and the event maximum depth for pelagic dives (based on the index of benthic diving behaviour, intra-depth zone; IDZ) carried out by Gentoo Penguin *Pygoscelis papua* breeding at the South End colony (New Island, Falkland/Malvinas Islands) during chick guard (December) in 2014. Details for the regression curve are given in **Table S4**





**Figure S15.** The relationship between the bottom time and the event maximum depth for benthic dives (based on the index of benthic diving behaviour, intra-depth zone; IDZ) carried out by Gentoo Penguin *Pygoscelis papua* breeding at the North End colony (New Island, Falkland/Malvinas Islands) during chick guard (December) in 2014. Details for the regression curve are given in **Table S4**





**Figure S16.** The relationship between the bottom time and the event maximum depth for pelagic dives (based on the index of benthic diving behaviour, intra-depth zone; IDZ) carried out by Gentoo Penguin *Pygoscelis papua* breeding at the North End colony (New Island, Falkland/Malvinas Islands) during chick guard (December) in 2014. Details for the regression curve are given in **Table S4**





**Figure S17.** Chlorophyll *a* concentration (4km, 8-day average [mg/m**3], 13m data) obtained from the Giovanni Ocean Color Time-Series analysis system of the National Aeronautics and Space Administration (NASA, USA). A) 3−19 Dec 2013, B) 3−19 Dec 2014

**
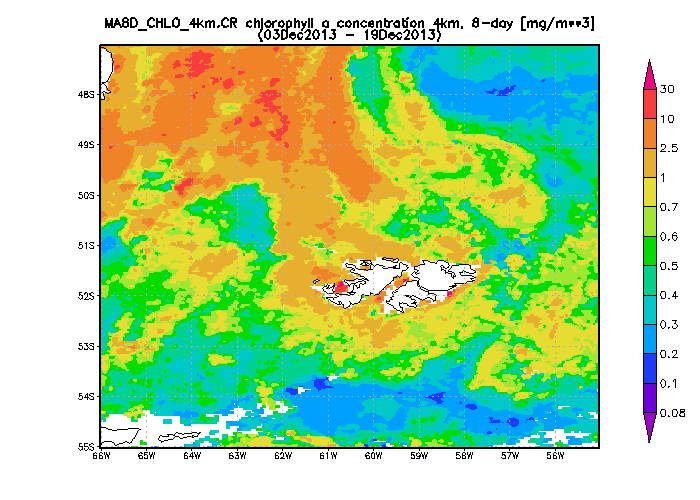
**


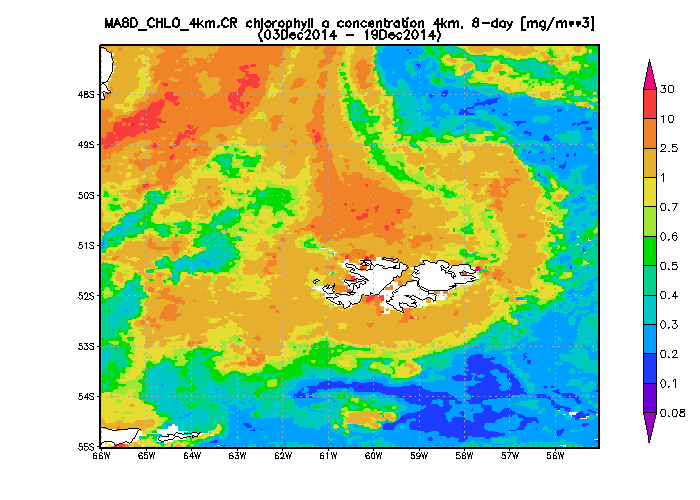

Supplement: Additional file 1: — Table S1. GAM investigating the sum of ODBAas a function of maximum dive depth. Table S2. Relationship between the sum of ODBA and maximum dive depth. Table S3. GAM investigating the bottom time as a function of event maximum depth. Table S4. Relationship between bottom time and event maximum depth. Figure S1. Depth zones. Figure S2. Distribution of depth during benthic (A) and pelagic (B) dives. Figure S3. Distribution in different depths of benthic (A) and pelagic (B) dives. Figure S4. Benthic dives example. Figure S5.Sum of ODBA versus maximum dive depth for benthic dives (South End, 2013). Figure S6. Sum of ODBA versus maximum dive depth for pelagic dives (South End, 2013). Figure S7. Sum of ODBA versus maximum dive depth for benthic dives (South End, 2014). Figure S8. Sum of ODBA versus maximum dive depth for pelagic dives (South End, 2014). Figure S9. Sum of ODBA versus maximum dive depth for benthic dives (North End, 2014). Figure S10. Sum of ODBA versus maximum dive depth for pelagic dives (North End, 2014). Figure S11. Bottom time versus event maximum depth for benthic dives (South End, 2013). Figure S12. Bottom time versus event maximum depth for pelagic dives (South End, 2013). Figure S13. Bottom time versus event maximum depth for benthic dives (South End, 2014). Figure S14. Bottom time versus event maximum depth for pelagic dives (South End, 2014). Figure S15. Bottom time versus event maximum depth for benthic dives (North End, 2014). Figure S16. Bottom time versus event maximum depth for pelagic dives (North End, 2014). Figure S17. Chlorophyll a concentration. (DOCX 2492 kb) [file 12983_2017_219_MOESM1_ESM.docx]
